# VISITATION SHELTERS FOR THE LONG-TERM CARE SETTING DURING THE PANDEMIC: AN EXPLORATORY STUDY

**DOI:** 10.1093/geroni/igad104.1853

**Published:** 2023-12-21

**Authors:** Michelle Porter, Shauna Mallory-Hill

**Affiliations:** University of Manitoba, Winnipeg, Manitoba, Canada; Faculty of Architecture, University of Manitoba, Winnipeg, Manitoba, Canada

## Abstract

In 2020, the province of Manitoba in Canada devised a plan to provide external visitation shelters for long-term care (LTC) facilities. The intent of the shelters was to alleviate the problems with visitor restrictions due to the COVID-19 pandemic. Dozens of visitation shelters, which were re-purposed shipping containers, were installed at facilities across the province. The purpose of this research was to: examine the experiences of the users through online surveys; make field measurements of the conditions (CO2 values, lighting, acoustics); and conduct an environmental scan of documents (government, media, etc.). Media and government documents provided insights into: design considerations; timing of installation/use; policies and procedures; how much they were utilized; as well as constraints. Survey findings from family/friends (n=20), LTC staff (n=9) and a resident (n=1) revealed that while many agreed that the shelters made a difference for their emotional well-being, many felt that the shelters did not support meaningful connections between residents and visitors. Furthermore, many respondents described the shelters as institutional/sterile and suggested that the décor should be improved to make them homier. Field measurements showed that the ventilation system ensured that CO2 values remained low, indicating that substantial fresh air exchange occurred. Acoustic values indicated that there could be challenges with residents hearing visitors. Lighting values demonstrated that color temperature was appropriate, but lux values were relatively low depending on the specific location in the shelter. Overall, this study was able to outline the advantages and disadvantages to using external visitation shelters in a pandemic situation.

